# Supplementary material for: Feeding Spodoptera exigua larvae with gut-derived Escherichia sp. increases larval juvenile hormone levels inhibiting cannibalism
Source: Commun Biol. 2023 Oct 26;6:1086. doi: 10.1038/s42003-023-05466-x (PMC10603045; doi:10.1038/s42003-023-05466-x)

Supplementary Figures

Figure S1

Standard qPCR curves that used to determine the bacterial load and *Spdoptera exigua* larval sex.

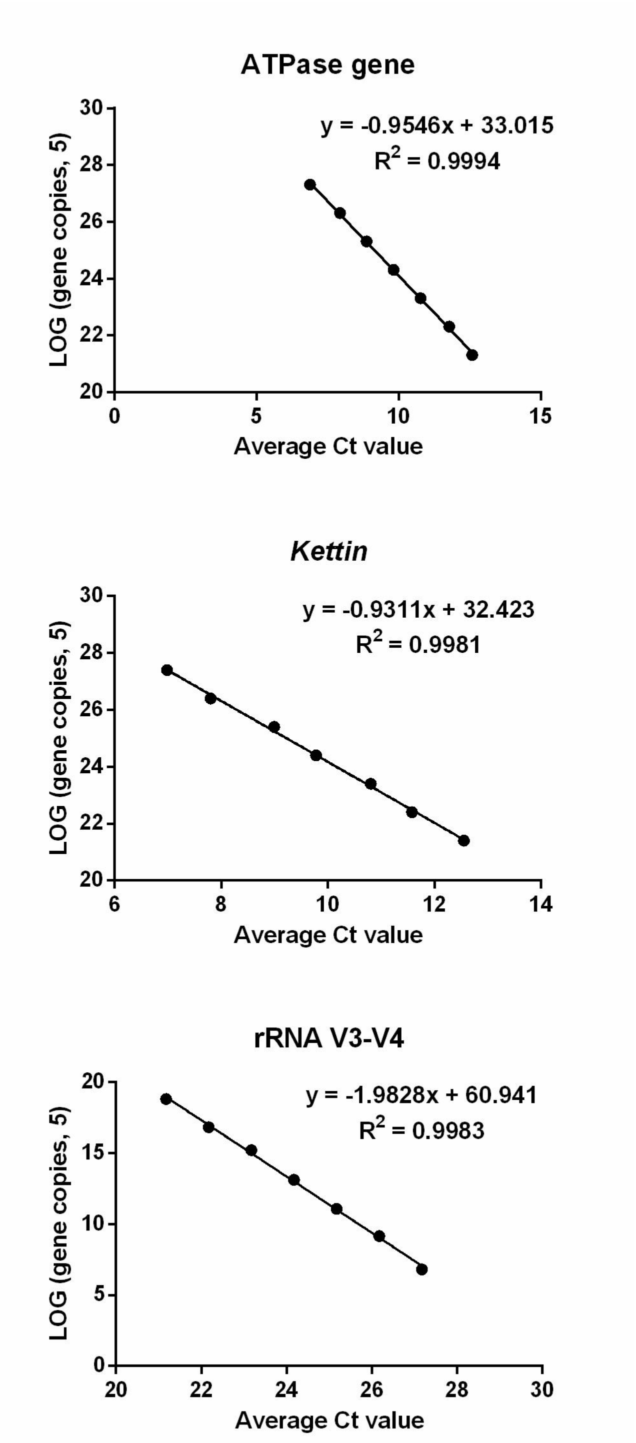

Figure S2

Venn diagram of OUTs from *S. exigua* larval midgut supplied with the five diets.

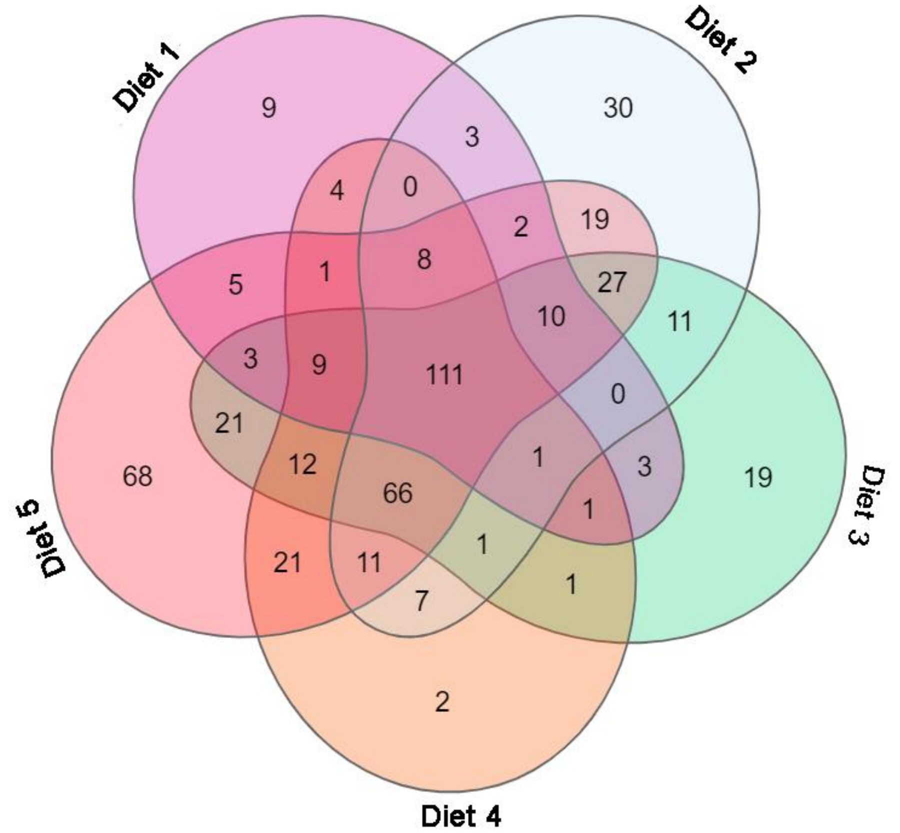

Figure S3

Isolation and identification of culturable bacteria from the gut of *S. exigua* larvae fed RW diet. A. The morphology of the bacterial colonies. B. The PCR products of 16S rRNA gene amplified from the DNA of the 15 bacterial isolates.

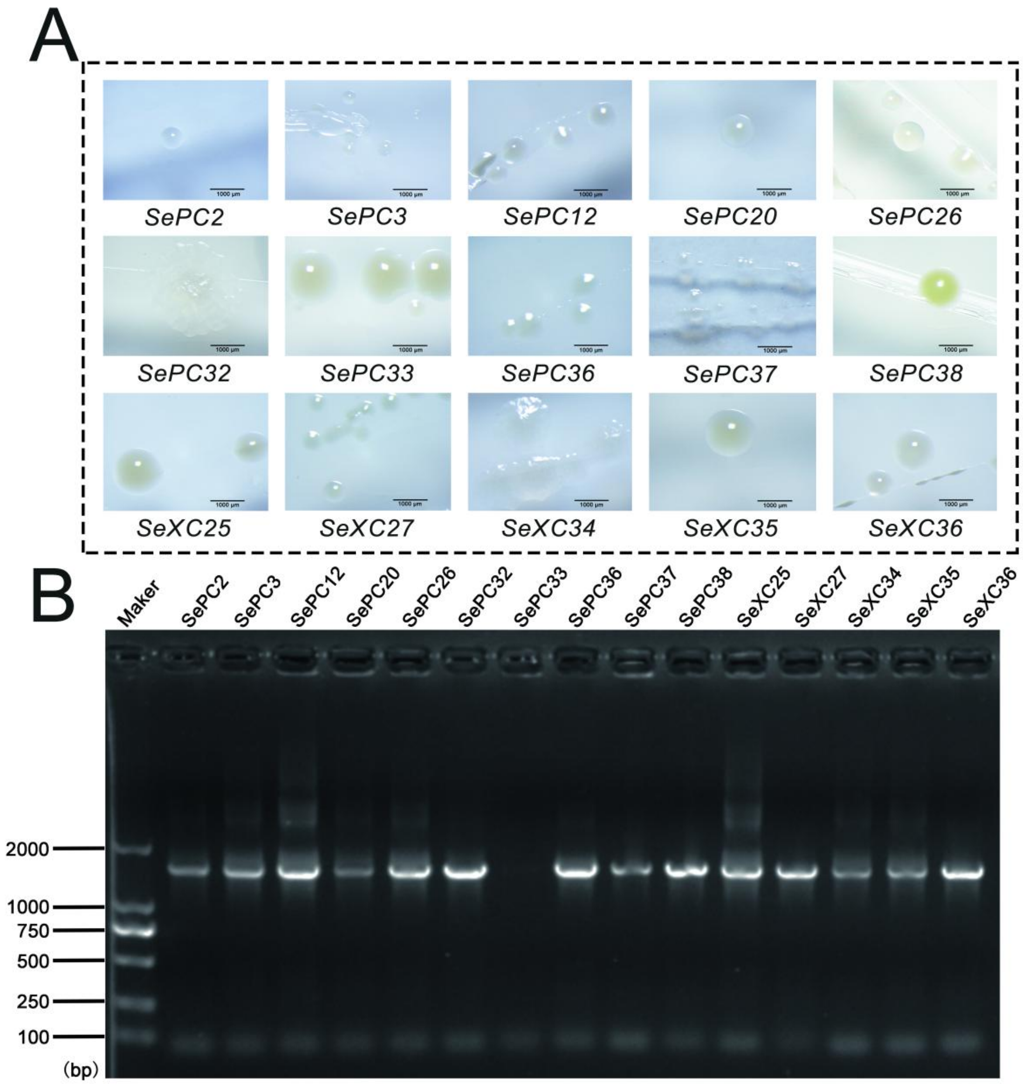

Figure S4

Neighbor-joining tree of gut microbiota of *S. exigua* and their closely related species based on partial sequence of the 16S rRNA gene.

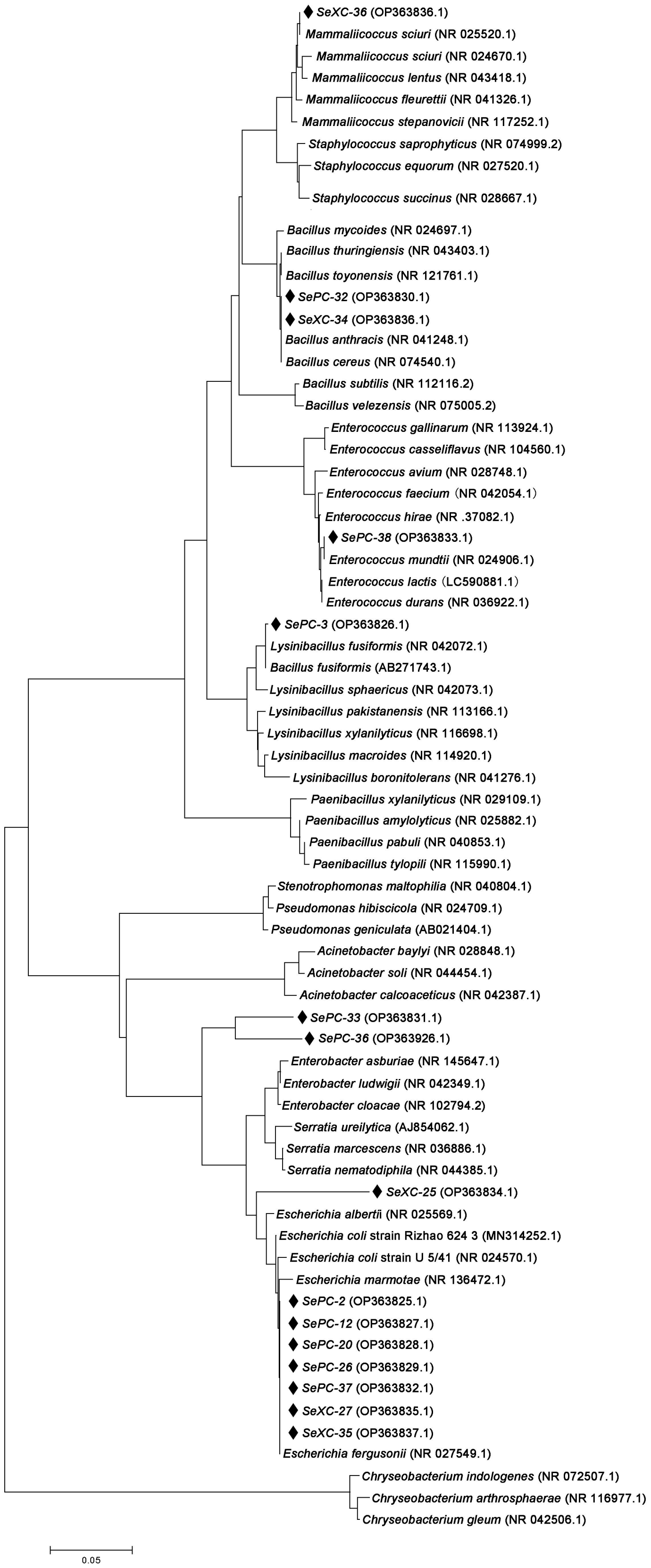

Figure S5

Nutrient composition of the six artificial diets used in the present study.

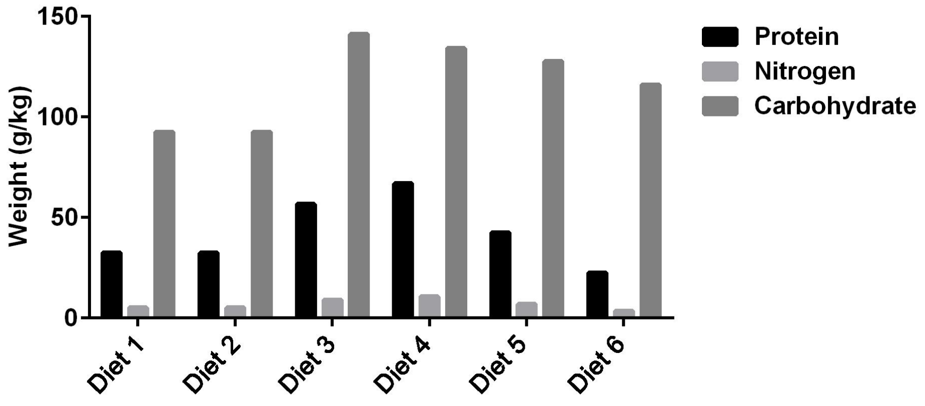

Figure S6

Standard curves used for insect hormone determination.

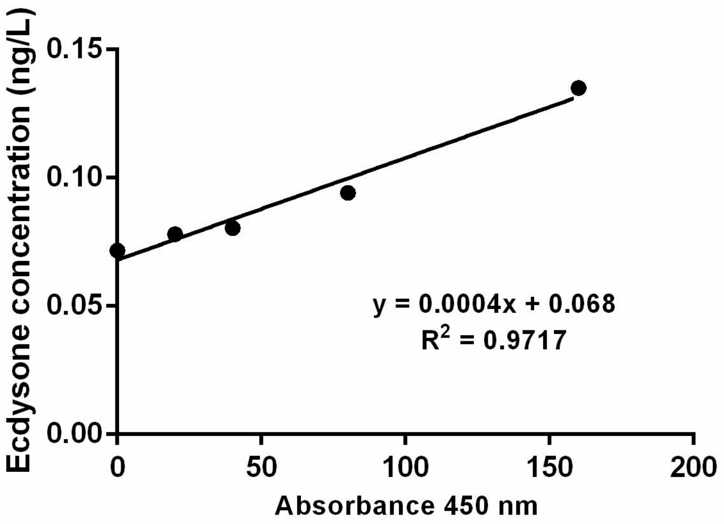

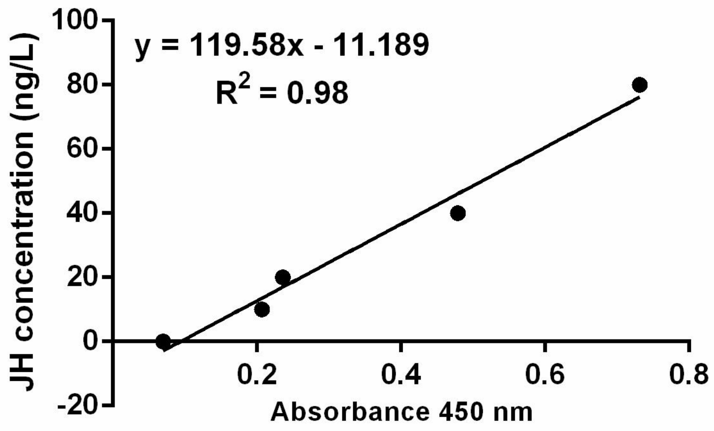

Supplement: Supplementary file 2 — Supplementary Figures [file 42003_2023_5466_MOESM2_ESM.pdf]
