# Supplementary material for: Feeding Spodoptera exigua larvae with gut-derived Escherichia sp. increases larval juvenile hormone levels inhibiting cannibalism
Source: Commun Biol. 2023 Oct 26;6:1086. doi: 10.1038/s42003-023-05466-x (PMC10603045; doi:10.1038/s42003-023-05466-x)
Supplement: Supplementary file 3 — Description of Additional Supplementary Files [file 42003_2023_5466_MOESM3_ESM.pdf]

Description of Additional Supplementary Files

File name: Supplementary Data 1

Description: The source data behind all the figures.

File name: Supplementary Data 2

Description: ANOVA of the daily cannibalism ratio of *S. exigua* larvae fed five different diets.

File name: Supplementary Data 3

Description: Summary of Illumina DNA reads generated from the 16S rRNA of *Spodoptera exigua* larval midgut.

File name: Supplementary Data 4

Description: Tag Splicing information.

File name: Supplementary Data 5

Description: OTUs in different samples.

File name: Supplementary Data 6

Description: Annotation of OTUs.

File name: Supplementary Data 7

Description: Relative abundance of OTUs in different samples at phylum level.

File name: Supplementary Data 8

Description: Relative abundance of OTUs in different samples at class level.

File name: Supplementary Data 9

Description: Relative abundance of OTUs in different samples at order level.

File name: Supplementary Data 10

Description: Relative abundance of OTUs in different samples at family level.

File name: Supplementary Data 11

Description: Relative abundance of OTUs in different samples at genus level.

File name: Supplementary Data 12

Description: Relative abundance of OTUs in different samples at species level.

File name: Supplementary Data 13

Description: OTU taxonomic assignments of Diet 1 vs Diet 3.

File name: Supplementary Data 14

Description: OTU taxonomic assignments of Diet 2 vs Diet 3.

File name: Supplementary Data 15

Description: OTU taxonomic assignments of Diet 4 vs Diet 3.

File name: Supplementary Data 16

Description: OTU taxonomic assignments of Diet 5 vs Diet 3.

File name: Supplementary Data 17

Description: OTUs in wilcox test of Diet 1 vs Diet 3.

File name: Supplementary Data 18

Description: OTUs in wilcox test of Diet 2 vs Diet 3.

File name: Supplementary Data 19

Description: OTUs in wilcox test of Diet 4 vs Diet 3.

File name: Supplementary Data 20

Description: OTUs in wilcox test of Diet 5 vs Diet 3.

File name: Supplementary Data 21

Description: Phenotypic characteristics and biochemical characteristice of bacterial isolates.

File name: Supplementary Data 22

Description: ANOVA of the daily cannibalism ratio of *S. exigua* larvae fed Diet 1 supplied with 15 bacterial isolates.

File name: Supplementary Data 23

Description: ANOVA of the daily cannibalism ratio of *S. exigua* larvae fed Diet 2 supplied with 15 bacterial isolates.

File name: Supplementary Data 24

Description: ANOVA of the daily cannibalism ratio of *S. frugiperda*, *H. armigera*, *H. assulta* larvae fed Diet 1 or Diet 2 supplied with *SePC-12* and *SePC-37*.
