# Supplementary material for: Feeding Spodoptera exigua larvae with gut-derived Escherichia sp. increases larval juvenile hormone levels inhibiting cannibalism
Source: Commun Biol. 2023 Oct 26;6:1086. doi: 10.1038/s42003-023-05466-x (PMC10603045; doi:10.1038/s42003-023-05466-x)
Supplement: Supplementary file 4 — Supplementary Data 1 [file 42003_2023_5466_MOESM4_ESM.zip › Supplementary Data 1/The source data behind Figure 4.docx]

All the produced reads for the bioinformatic analysis of Figure 4 are available on GitHub (https://github.com/FiFiFishsh/For-mBio-20221105)

All the data used for the figure establish were provided in SUPPLIMENTARY TABLES (Table S2-S19).
